# The Impact of cDNA Normalization on Long-Read Sequencing of a Complex Transcriptome

**DOI:** 10.3389/fgene.2019.00654

**Published:** 2019-07-23

**Authors:** Nam V. Hoang, Agnelo Furtado, Virginie Perlo, Frederik C. Botha, Robert J. Henry

**Affiliations:** ^1^College of Agriculture and Forestry, Hue University, Hue, Vietnam; ^2^Queensland Alliance for Agriculture and Food Innovation, The University of Queensland, St. Lucia, QLD, Australia; ^3^Sugar Research Australia, Indooroopilly, QLD, Australia

**Keywords:** isoform sequencing, transcriptome normalization, transcript enrichment, normalization impact, sugarcane transcriptome, polyploid transcriptome

## Abstract

Normalization of cDNA is widely used to improve the coverage of rare transcripts in analysis of transcriptomes employing next-generation sequencing. Recently, long-read technology has been emerging as a powerful tool for sequencing and construction of transcriptomes, especially for complex genomes containing highly similar transcripts and transcript-spliced isoforms. Here, we analyzed the transcriptome of sugarcane, a highly polyploidy plant genome, by PacBio isoform sequencing (Iso-Seq) of two different cDNA library preparations, with and without a normalization step. The results demonstrated that, while the two libraries included many of the same transcripts, many longer transcripts were removed, and many new generally shorter transcripts were detected by normalization. For the same input cDNA and data yield, the normalized library recovered more total transcript isoforms and number of predicted gene families and orthologous groups, resulting in a higher representation for the sugarcane transcriptome, compared to the non-normalized library. The non-normalized library, on the other hand, included a wider transcript length range with more longer transcripts above ∼1.25 kb and more transcript isoforms per gene family and gene ontology terms per transcript. A large proportion of the unique transcripts comprising ∼52% of the normalized library were expressed at a lower level than the unique transcripts from the non-normalized library, across three tissue types tested including leaf, stalk, and root. About 83% of the total 5,348 predicted long noncoding transcripts was derived from the normalized library, of which ∼80% was derived from the lowly expressed fraction. Functional annotation of the unique transcripts suggested that each library enriched different functional transcript fractions. This demonstrated the complementation of the two approaches in obtaining a complete transcriptome of a complex genome at the sequencing depth used in this study.

## Introduction

Advances in sequencing technologies in recent years have allowed a great amount of transcriptomic data to be generated within a relatively short time at an affordable price. Short-read technologies have been used for transcriptome sequencing and transcript expression across a broad transcript dynamic range. However, the quality of transcriptomic analyses including transcript profiling and differential expression analysis greatly relies on the quality of the available reference transcriptome (Brown et al., 2017). Studies on the dynamic changes in the transcriptome and gene expression corresponding to the plant responses to internal and environmental signals, which are the keys to understanding of plant growth and developmental processes, can be facilitated by having a high-quality reference transcriptome (e.g., complete full-length sequences generated from long-read technologies), in combination with deep sequencing data (e.g., from RNA-Seq). It is still challenging, in most species, to obtain a high-quality and complete reference transcriptome, which represents the different isoforms of all of the gene content. In higher plants, analysis is complicated by up to 70% of intron-containing genes producing different transcript isoforms through alternative splicing, resulting in translation into different functional proteins (Nilsen and Graveley, 2010; Chamala et al., 2015; Lee and Rio, 2015). The assembly and prediction of the highly similar transcript isoforms sharing the same exons based on short-read data still remain difficult, especially for species without a reference genome or with incomplete genome sequences (Martin and Wang, 2011). Recently, long-read sequencing technologies have been emerging as a potential strategy to recover the complexity of the transcriptome without the need of assembly and have been applied to several plant species (Dong et al., 2015; Abdel-Ghany et al., 2016; Wang et al., 2016; Cheng et al., 2017; Clavijo et al., 2017; Hoang et al., 2017a; Li et al., 2017b; Mascher et al., 2017; Wang et al., 2017b; Wang et al., 2018; Workman et al., 2018). These results illustrate the advance of long-read sequencing technologies in capturing and sequencing of alternative spliced transcript isoforms, providing more accurate evidence of transcript length, alternative splicing events, and polyadenylation; improving the reference transcriptome annotation; and allowing more accurate transcript profiling when coupled with splicing-aware mapping algorithms.

Conventionally, for transcriptome library preparation, a sample comprised of multiple cells isolated from the tissue/organ being studied is normally used for RNA extraction. When sequenced, this protocol tends to work well for medium to highly or ubiquitously expressed transcripts which are normally represented in high copy number and are predominant in the transcriptome. However, transcripts of genes that are expressed at low levels or expressed in a small fraction of the cell types of a tissue and/or at certain phases of development can comprise a small fraction of the total transcripts of an extracted RNA sample. As a result, the predominant transcripts have a higher probability of being sequenced multiple times compared to those transcripts expressed at low levels, which might not be sequenced at all considering the relatively low throughput (at an affordable cost) of most recently available long-read transcriptome sequencing platforms including the PacBio Iso-Seq (Eid et al., 2009), the Illumina Tru-Seq Synthetic Long-Read Technology (Voskoboynik et al., 2013), and the Oxford Nanopore Technology (Clarke et al., 2009; Jain et al., 2016). Many of the transcripts expressed at low levels are important developmental regulators (including coding and non-coding transcripts) which could be crucial in the understanding of plant developmental and biological processes and tend to be expressed at low levels and in a tissue-specific manner (Mita et al., 2003; Bogdanova et al., 2008). To improve the coverage of rare transcripts and reduce the abundance of highly expressed transcripts in the library preparation step for transcriptome construction, normalization of cDNA was developed (Shcheglov et al., 2007). Among the methods that have been developed, cDNA normalization employing a Duplex-specific nuclease enzyme (DSN) (Zhulidov et al., 2004; Zhulidov et al., 2005; Bogdanova et al., 2010; Bogdanova et al., 2011) has been widely used to increase the gene discovery rate and reduce the sequencing redundancy by equalizing the abundance of transcripts of different expression levels. This protocol has been applied successfully to long-read transcriptome sequencing for not only plant species but also animal species including Danshen (Zhichao et al., 2015), sugarcane (Hoang et al., 2017a), coffee (Cheng et al., 2017), pineapple (Wang et al., 2017a), hookworm (Magrini et al., 2018), and fungus (Cook et al., 2019). On the other hand, the use of non-normalized cDNA libraries for long-read sequencing has been employed in several other studies (Dong et al., 2015; Abdel-Ghany et al., 2016; Wang et al., 2016; Li et al., 2017a; Li et al., 2017b; Wang et al., 2017b; Anvar et al., 2018; Wang et al., 2018; Wen et al., 2018; Minio et al., 2019; Qiao et al., 2019). However, the impact of normalization on long-read transcriptome sequencing has not been studied.

We now report the use of the PacBio Iso-Seq data, generated for the transcriptome of the highly polyploid sugarcane genome in an earlier study (Hoang et al., 2017a), to assess the impact of cDNA library experimental normalization on long-read sequencing. This was achieved by comparing two different approaches of cDNA library preparation, with and without the normalization step to provide insights into the differences between the two approaches employed in cDNA library preparation. This knowledge will be useful in future long-read sequencing projects allowing improved sugarcane transcriptome construction, gene expression studies, and genome annotation. The sugarcane genome is amongst the most complex plant genomes due to the high level of polyploidy, aneuploidy, heterozygosity, and interspecificity (Grivet and Arruda, 2002; Thirugnanasambandam et al., 2018). The monoploid genome size is estimated to be around 1 Gb (composed of 10 basic chromosomes). However, complexity resides in the distinct recombination of aneuploid and homo(eo)logous chromosomes, originally from two progenitor species, that results in a total chromosome number typically between 100 and 130, and around 10 Gb in size (Simmonds, 1976; Sreenivasan et al., 1987; D’Hont et al., 1996; Grivet et al., 1996; D’Hont and Glaszmann, 2001). This complexity makes it extremely challenging to obtain a reference sequence for sugarcane. The recently released sugarcane mosaic monoploid genome sequence representing 382 Mb of gene-rich regions of sugarcane based on the sorghum genome (Garsmeur et al., 2018) was also exploited in this study, to evaluate the transcriptome data.

## Materials and Methods

### Plant Materials and RNA Extraction

Sugarcane materials and DNA extraction were described previously (Hoang et al., 2017a). In brief, a total of 22 sugarcane cultivars varying in fiber and sugar content were provided by Sugar Research Australia. Leaf, internodal, and root tissues representing different developmental stages were collected and used for RNA extraction. RNA was extracted for each of the leaf, internodal, and root samples, before pooling to form one single RNA sample representing all cultivars and all developmental stages. Only samples of a RIN > 7.5 were used for further analysis.

### cDNA Library Preparation and Sequencing

**Figure 1:**
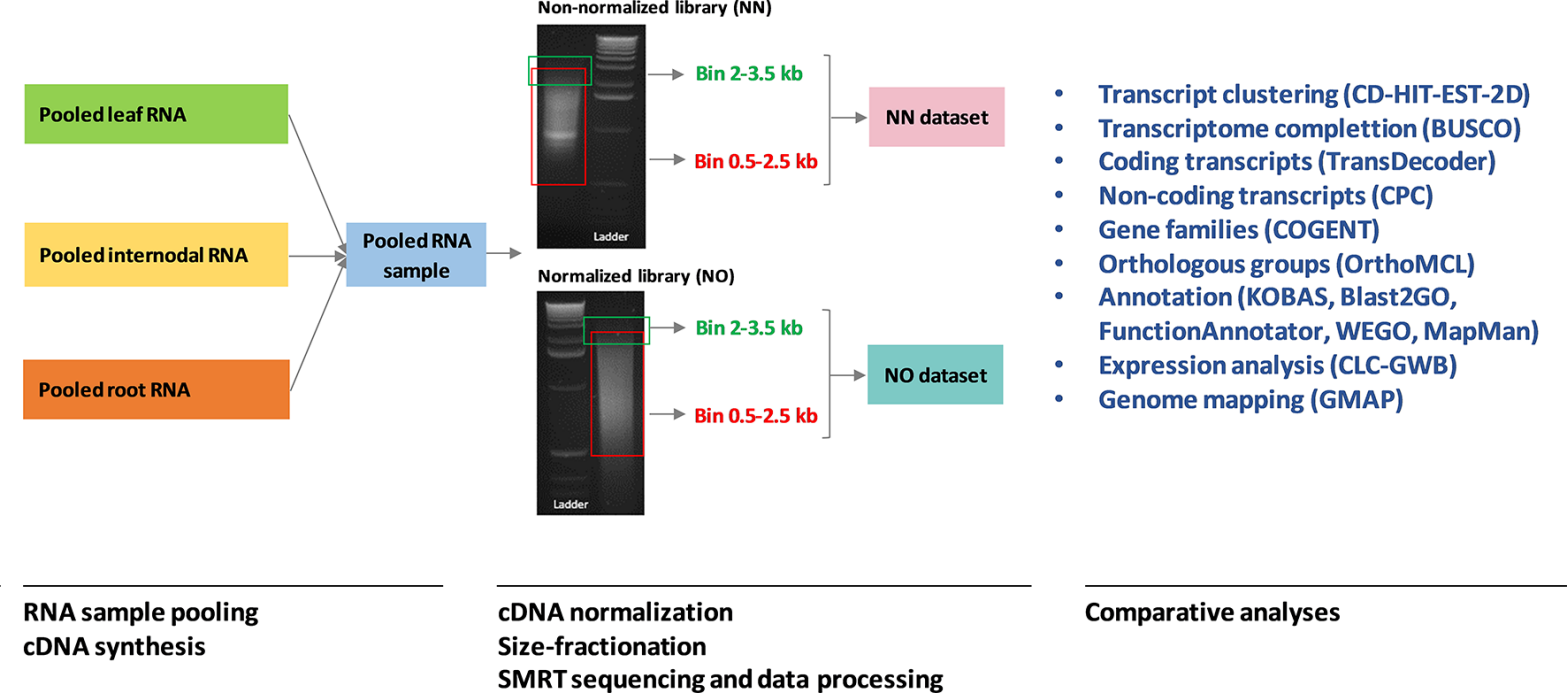
A summary of the analysis workflow used in this study. The RNA sample pooling, cDNA synthesis, normalization, size fractionation, and sequencing data processing were previously reported in Hoang et al. (2017a) in which the data from the two libraries were combined and analyzed. The original gel images of the sugarcane non-normalized and normalized cDNA libraries resolved on 1.2% aragose were adapted from Hoang et al. (2017a). NN denotes the sugarcane non-normalized PacBio Iso-Seq isoforms while NO denotes the sugarcane normalized PacBio Iso-Seq isoforms.

The cDNA library preparation followed the PacBio Iso-Seq guidelines on an aliquot of 1 μg pooled RNA, as previously described (Hoang et al., 2017a). In summary, for the non-normalized library, first strand cDNA was synthesized using a SMARTer PCR cDNA Synthesis Kit (Clontech, Takara Bio Inc., Shiga, Japan) and amplified for 18 cycles using a KAPA HiFi PCR Kit (Kapa Biosystems, Boston, USA). The resultant cDNA library was size-fractionated employing the BluePippin system (Sage Science) into four different bins, 0.5–2.5, 2–3.5, 3–6, and 5–10 kb. For the normalized library, 1 μg of the non-normalized cDNA library was normalized following Trimmer-2 (Evrogen, Moscow, Russia) using 1U enzyme DSN and subsequently amplified for 18 cycles using KAPA HiFi enzyme from the KAPA HiFi PCR Kit. The normalized cDNA library was size-fractionated into two bins of 0.5–2.5 and 2–3.5 kb. **Figure 1** shows PCR amplification of the two libraries before size fractionation, previously reported in Hoang et al. (2017a), in which the normalized library showed a smoother transcript length distribution without any visible bands compared to that from the non-normalized library. After size fractionation, both libraries were sequenced in six SMRT cells (corresponding to six bins) on a PacBio RS II instrument at the Ramaciotti Centre for Genomics, the University of New South Wales, NSW, Australia. For comparison in this study, we included only data from two bins of 0.5–2.5 and 2–3.5 kb from the two libraries for further analysis.

### Iso-Seq Read Processing

The read data processing followed steps previously described (Hoang et al., 2017a) with some modifications. PacBio Iso-Seq reads were analyzed and processed by the SMRT analysis package ver2.3.0 (PacBio) to retain only full length (FL) and high- and low-quality transcript sequences. The FL reads were defined as those having the 5’ prime-, 3’ prime adapters and a polyA tail (PacBio, 2018), which subsequently were removed during the read processing. The FL low-quality reads were defined as those FL transcript with relative low numbers of supporting reads during the self-correction. We retained both FL high-quality and low-quality transcripts and concatenated them into one single file for downstream analysis. Additionally, sequences containing polyA and primer adapter within them due to sequencing errors were identified by further searched and trimmed using CLC Genomics Workbench ver10 (CLC Bio-Qiagen, Aarhus, Denmark).

### Bioinformatics Analysis

Sequences of 99% identity and 99% length coverage were collapsed into one single cluster by CD-HIT-EST from the CD-HIT package ver4.6.8 (Fu et al., 2012) with the default settings and following parameters: -c 0.99 -n 10 -aL 0.99. These stringent parameters were employed to ensure that only duplicate sequences were removed, since the longest sequences normally retained by CD-HIT are not always the best sequences (assessed by protein metrics, e.g., ORF length and count). Comparing between bins and combined datasets was done by using CD-HIT-EST-2D from the CD-HIT package, with the following parameters: -c 0.8 -n 10 and other default settings. The program performed quantitative two-way comparison, which allowed comparison of the sequence abundance and identity from the two datasets.

Transcriptome completion was assessed by Benchmarking Universal Single-Copy Orthologs (BUSCO) ver3.0.2b employing 303 orthologous groups derived from 90 representative eukaryotic species (Simão et al., 2015). Coding sequences (ORFs of minimum 100 aa) were extracted by TransDecoder ver5.3.0 (TransDecoder, 2018). Orthologous gene pair was predicted by OrthoMCL 5 (Li et al., 2003) with default settings, BLASTP cutoff e-value = -5 and similarity 50% match. Transcripts were assigned to putative gene families by the COding GENome reconstruction Tool ver3.3 (Cogent) pipeline developed for PacBio Iso-Seq data (Cogent, 2018). For short-read validation and expression analysis, Illumina RNA-Seq reads (150 bp paired end) from leaf and root tissues (Mason et al., unpublished) and stalk tissue (Hoang et al., 2017b) were mapped against the total and unique transcripts using RNA-Seq tool in the CLC Genomics Workbench ver10, with the following parameters: length fraction (0.5), similarity fraction (0.8), using EM estimation, and other default settings. Three biological replicates were used for each of the tissue types. The expression level was measured as total raw counts and normalized as Reads per kilobase per million mapped reads (RPKM) (Mortazavi et al., 2008), to allow comparison between the two groups of transcript datasets of different length and different sequences. Long noncoding transcripts were predicted using Coding Potential Calculator (CPC) ver0.9-r2 (Kong et al., 2007), against UniRef_90 (UniProt, 2018). Transcript annotation was done by comparing against the sorghum and *Arabidopsis* genomes using KOBAS ver3.0 (Xie et al., 2011) with an e-value of 1e-5, and then against the NCBI non-redundant (NR) protein database and Gene Ontology (GO) database using LAST (Frith et al., 2010) and BLAST2GO (Conesa and Gotz, 2008), respectively, through FunctionAnnotator (Chen et al., 2017). GO terms from each datasets were enriched, compared, and plotted using the Web Gene Ontology Annotation Plot (WEGO) ver2.0, GO version 2018-03-01 (Ye et al., 2006; Ye et al., 2018). Further annotation was done using the Mercator sequence annotator 4 ver1.0 (Lohse et al., 2014) and analyzed in MapMan ver3.6.0RC1 (Thimm et al., 2004; Usadel et al., 2009) to dissect the unique fractions of the two libraries. For genome-wide distribution, transcripts were aligned against the sugarcane monoploid genome sequences [Garsmeur et al. (2018), downloaded from CIRAD (2018)] and sorghum genome ver3 (Phytozome v12.1.6, 2018) by GMAP ver2018-07-04 (Wu and Watanabe, 2005) with 80% coverage and 80% identity thresholds and visualized using Circos (Krzywinski et al., 2009).

Venn diagrams were created using the online Venn tools (Draw Venn Diagram, 2016; InteractiVenn, 2017). Statistical analyses and graphical presentation using R packages including ggplot2 (Wickham, 2009) and reshape2 (Wickham, 2007) were run in R ver3.4.2. Analyses in CLC Genomics Workbench ver11 were conducted using QAAFI Bioinformatics Infrastructure, at the University of Queensland, Australia. Analyses using command-line packages were performed at the HPC clusters, the University of Queensland, Australia (Research Computing Centre, 2018). A summary of the analysis workflow used in this study, from RNA sample pooling to comparative analyses between the two dataset, is provided in **Figure 1**.

### Data Availability

The raw data supporting the conclusions of this study have been previously deposited in NCBI SRA database under BioProject PRJNA356226, accession numbers from SRR5259105 to SRR5259110. The RNA-Seq read data for leaf and root tissues can be downloaded in NCBI Study Accession Number SRP152893. All other relevant data that supports the findings in this study are included within the article and supplementary files.

## Results

### Iso-Seq Data Clustering and Comparison Suggest That Normalization Removes Longer Transcripts and Recovered More Short Transcripts

**Figure 2:**
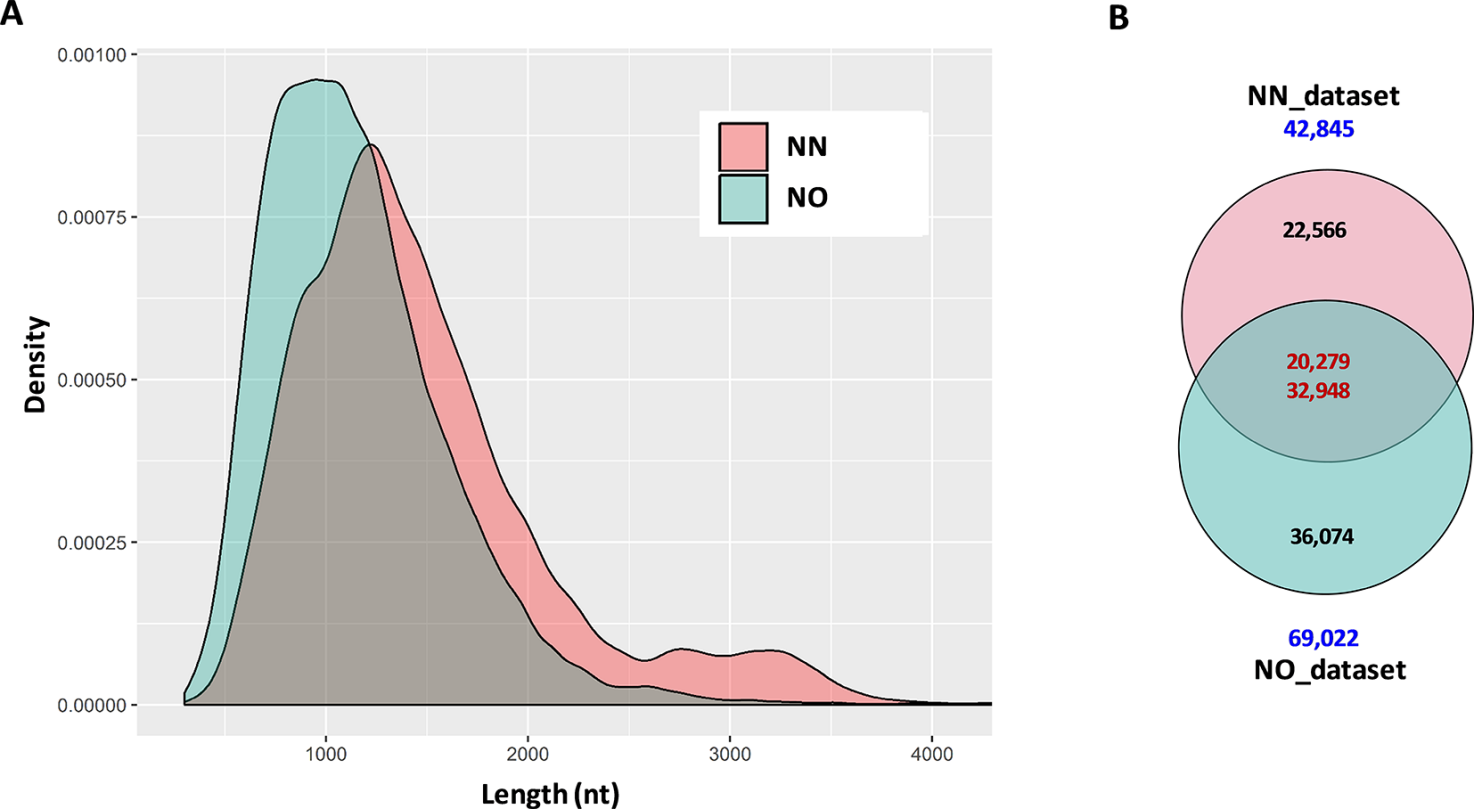
Summary statistics of data and comparison between datasets. **(A)** Length distribution of distribution of combined data from two bins 0.2–2.5 kb and 2–3.5 kb from each library. For visualization, only transcripts ≤4 kb were used. **(B)** Two directional comparison between sequences from the two libraries by CD-HIT-EST-2D. The upper number in the intersection of the Venn diagrams represents the transcripts from the non-normalized dataset, while the lower number is from the normalized dataset. NN, sugarcane non-normalized PacBio Iso-Seq isoforms; NO, sugarcane normalized PacBio Iso-Seq isoforms.

The initial data processing was performed for two datasets obtained from non-normalized and normalized cDNA libraries (hereafter, referred to as NN and NO, respectively). Each library had two bins of 0.5–2.5 kb and 2–3.5 kb. The NN dataset has 15,014 high-quality (HQ) and 7,773 low-quality (LQ)-FL transcript isoforms (transcripts) in the 0.5–2.5 kb bin; 11,880 HQ; and 8,309 LQ in the 2–3.5 kb bin. This resulted in the total number of pooled transcript isoforms in the NN dataset being 42,976 sequences. Similarly, the NO dataset had 17,597 HQ- and 8,147 LQ-FL transcript isoforms in the 0.5–2.5 kb bin; 27,703 HQ; and 15,624 LQ in the 2–3.5 kb bin. The total number of transcripts obtained for the normalized library was 69,071. The HQ- and LQ-FL transcripts in each dataset were combined into single files for further analysis. To reduce the redundancy, we first collapsed all sequences of 99% similarity and 99% overlapped length by CD-HIT-EST, which resulted in 131 sequences in the NN dataset and 49 sequences in NO dataset being removed. The sequence summary statistics for each bin in the two datasets are presented in **Table 1**. The total transcripts after CD-HIT clustering was 42,845 and 69,022 for the NN and NO datasets, respectively. The transcript length ranged from 302 nt to 12,828 nt (N50: 1,580 nt) for NN dataset, and 301 nt to 13,769 nt (N50: 1,251 nt) for the NO dataset. The length distribution of the two dataset is shown in **Figure 2A**.

**Table 1:** Summary statistics of the two final datasets of non-normalized and normalized libraries.

	NN dataset	NO dataset
Total sequences	42,845	69,022
Total bases (nt)	63,779,896	79,917,822
Average length (nt)	1,489	1,158
Median length (nt)	1,347	1,084
N50 (nt)	1,580	1,251
Min length (bp)	302	301
Max length (bp)	12,828	13,769
GC content (%)	52.8	50.1

The first question we asked was how many of the total transcripts in the two libraries were common and how many were recovered during the normalization that were not present in the NN library. To get a general idea of the difference between the two datasets, we compared them in two different ways, comparing bins of the same length and comparing total pooled data from each of the libraries. This was done by using the two-directional comparison (CD-HIT-EST-2D) at a sequence identity threshold of 80%. When the two bins of 0.5–2.5 kb were compared, a total of 7,661 transcripts in the NN dataset were found to be similar to 9,981 transcripts in the NO dataset, which accounted for 33.6% and 38.8% of the respective datasets. Similarly, a total of 7,513 transcripts in the 2–3.5 kb bin of the NN dataset were found to be similar to 14,954 transcripts in the 2–3.5 kb bin of the NO dataset, which accounted for 37.2% and 34.5% of the respective datasets. When the pooled datasets representing all the sequences that were captured in both 0.5–2.5 and 2–3.5 kb bins from each library were compared, we identified 20,279 transcripts (47.3% of the NN dataset) and 32,948 (47.7% of the NO dataset) were similar at 80% identity, while 22,566 and 36,074 transcripts were unique to NN and NO datasets, respectively (**Figure 2B**). These results indicate that while the two libraries included many of the same transcripts, and cDNA normalization led to the loss of longer transcripts and the gain of a large number of unique shorter transcripts. The NN library recovered a wider range of transcript length with more longer transcripts of above 1.25 kb, while the NO library recovered more transcripts in total, especially those of length of below 1.25 kb.

### Combining the Two Libraries Results in a Better Sugarcane Transcriptome Completeness

**Figure 3:**
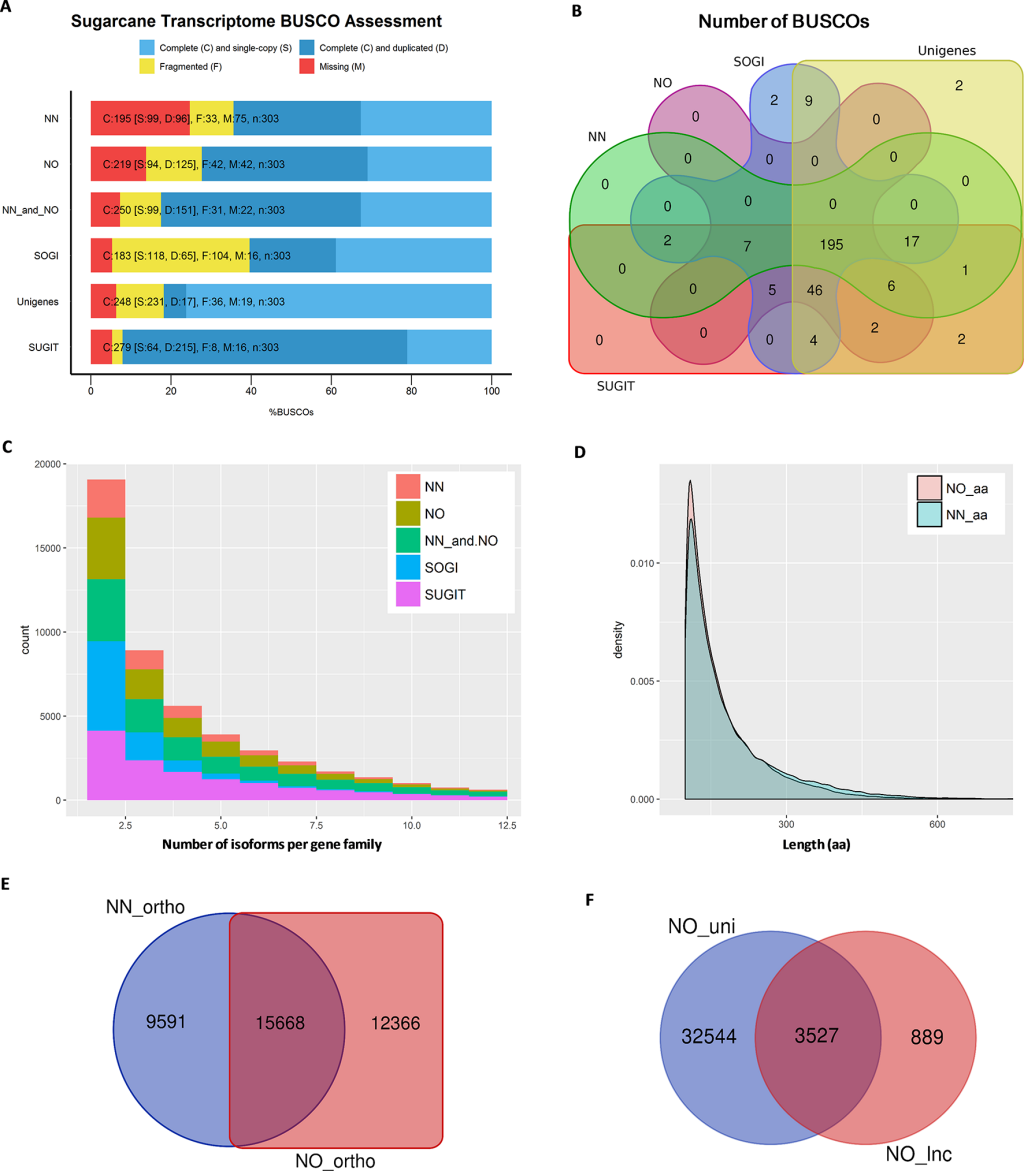
Transcriptome quality assessment *via* BUSCO, OrthoMCL, and Cogent packages. **(A)** BUSCO completeness assessment of two datasets, combined data and three reference transcriptome databases, SoGI, unigenes, and SUGIT. In the bar charts, C, S, D, F, and M denote complete, single, duplicate, fragmented, and missing BUSCOs. **(B)** Venn diagram showing BUSCOs recovered in each of datasets. **(C)** Number isoforms per gene family identified by Cogen pipeline. **(D)** Length distribution of extracted ORF sequences from the two datasets. **(E)** Venn diagram showing a comparison of orthologous groups between two datasets. **(F)** Long noncoding transcripts identified in the NO dataset (NO_lnc) compared against the unique fraction of transcripts from the NO dataset (NO_uni). NN, sugarcane non-normalized PacBio Iso-Seq isoforms; NO, sugarcane normalized PacBio Iso-Seq isoforms; SoGI, *Saccharum officinarum* gene indices; SUGIT, sugarcane Iso-Seq transcriptome; aa denotes amino acid.

We then estimated how complete each of the datasets was in terms of representing the sugarcane transcriptome. For this purpose, we employed the BUSCO package to count the number of essential single copy orthologs, which should be included in a good representative transcriptome of any eukaryote species (Simão et al., 2015). This completeness assessment used the eukaryotic lineages datasets “eukaryota_odb9,” which consists of 303 BUSCO orthologous groups derived from 90 selected representative species. The results showed that the NN dataset recovered 64.4% complete BUSCOs, 10.9% fragmented BUSCOs (totaling 75.3% detected sequences), and 24.7% missing BUSCOs, while the NO dataset included more BUSCOs having 72.3% complete, 13.9% fragmented (totaling 86.2%), and 13.8% missing BUSCOs. We checked the transcriptome completeness when both datasets (four bins) were combined, and it was improved to 82.5% complete and 10.2% fragmented (totaling 92.7%) and reduced the missing BUSCOs to only 7.3%. Additionally, when all bins from the two datasets including two upper non-normalized bins 3–6 kb and 5–10 kb (six bins) which were not used in our comparison, the BUSCO completeness was 85.5% complete and 8.9% fragmented (totaling 94.4%), and missing BUSCOs was 5.6%. All BUSCO analysis is summarized in **Figure 3A** and **Supplementary Table 1**. To allow further evaluation, we also compared the BUSCO performance of the two datasets with that of three reference transcriptomes, the SoGI (*Saccharum officinarum* gene index) (SoGI, 2017), unigenes from transcriptome reported in Cardoso-Silva et al. (2014), and the SUGIT, which was the combined dataset of NN and NO corrected by Illumina short-reads (Hoang et al., 2017a). Additionally, the CDS sequences from the new sugarcane monoploid genome (Garsmeur et al., 2018) were also compared. The SoGI and SUGIT had the lowest missing BUSCOs (5.3%), while unigenes also represented well the sugarcane transcriptome showing good BUSCO assessment (missing only 6.3% BUSCOs), and the sugarcane CDS sequences showed a higher missing rate compared to other datasets in the reference group (74.2% complete, 6.6% fragmented, totaling 80.8%, and 19.2% missing BUSCOs). **Figure 3B** shows a comparison of the total number of complete and fragmented BUSCOs of the NN and NO datasets against the reference groups. Between the two datasets, the common BUSCOs were 208, and more unique BUSCOs were detected in NO dataset (53) than that in the NN dataset (20). Among the reference group, it was found that 13 BUSCO were only detected in the SoGI and unigenes, including nine common, two were unique to the SoGI, and two were unique to the unigene dataset. The SUGIT included all BUSCOs detected in the two NN and NO datasets, and additional six BUSCOs (two BUSCOs when all six bins were included and compared), which indicates that the short-read correction improved that transcript quality, as suggested previously (Hoang et al., 2017a). Collectively, the results indicate that there was a proportion of the sugarcane transcriptome that was not represented in both datasets, and among the two, the NO dataset exhibited a higher BUSCO completion, suggesting that the normalization helped in recovered more transcripts in the sugarcane transcriptome in this case. The low completeness of the separate datasets is likely to be attributable to the relatively low sequencing depth by PacBio Iso-Seq in the current study, and pooling of the data from both libraries significantly improved the completeness by including more and different transcript isoforms. The total number of non-redundant transcript isoforms in the combined data of NN and NO was 111,867 (N50 = 1,382 nt), while the total real number of isoforms in the samples studied, which were derived from 22 genotypes, are unknown and could be more than that. Increasing the sequencing depth is likely to improve the completion of the transcriptome, especially for a very complex sugarcane transcriptome.

### Normalization Increases the Gene Discovery Rate by Including More Gene Families and Orthologous Groups

Next, we predicted the number of putative gene families included in each dataset using Cogent ver3.3. This package was designed for Iso-Seq data to find gene families and reconstruct the coding genome for a species with an incomplete genome sequence like sugarcane, based on the k-mer similarity to partition full-length coding sequences into gene families (Cogent, 2018). It was found that, of the total 42,845 transcripts in the NN dataset, 25,786 transcripts were clustered into 5,818 gene families and 16,685 orphan single-isoform genes, while the rest were classified as chimeras. Similarly, of the total 69,022 transcripts in the NO dataset, 39,283 transcripts were grouped into 9,783 putative gene families and 29,124 putative single-isoform genes. When the NN and NO datasets were combined, the total gene families were 13,276 (from 76,361 transcripts) and the single-isoform genes were 32,919. The gene families obtained for SoGI and SUGIT were 9,915 and 14,189, respectively. The unigene dataset contains only Trinity-based primary isoforms and was not included in this comparison. A summary of Cogent gene family prediction is presented in **Figure 3C** and **Supplementary Table 2**. The average number of isoforms calculated per gene family was 1.89 for NN, 1.76 for NO, 2.37 for combined NN and NO, 1.85 for SoGI, and 3.35 for SUGIT. When only the transcripts that clustered into multiple-isoform gene families were considered, the average transcript isoforms per family were 4.43 for NN, 4.02 for NO, 5.75 for combined dataset, 2.66 for SoGI, and 5.53 for SUGIT. The results suggest that, compared to the NN library, the NO library had an average less isoforms per gene family; however, it recovered more gene families, which could be attributed to the normalization of cDNA library resulting in more rare transcript isoforms being included, as shown in the previous section. The greater number of transcripts per gene family in the NN dataset could be that the data included a deeper sequencing depth for those highly abundant transcripts that captured in the non-normalized library.

We also predicted the number of orthologs in each of the libraries using OrthoMCL ver5 program. The open reading frames (ORFs) were extracted from transcripts using TransDecoder ver5.3.0, and the longest ORFs (min 100 aa) were retained for orthologous gene prediction. Consistent with the previous observation, the NN dataset produced 105,605 ORF sequences (99–1,121 aa, N50: 196 aa), while the NO dataset had 117,101 ORF sequences (99–2,663 aa, N50: 176 aa) (**Figure 3D**). The total numbers of transcripts with an ORF of at least 100 aa were 39,975 (∼93%) and 57,990 (∼84%) for the NN and NO datasets, respectively. A total of 39,936 transcripts were assigned to 25,259 orthologous groups in the NN dataset, compared to 57,953 transcripts and 28,034 orthologous groups from the NO. There were 7,917 and 8,312 protein sequences assigned to “NO_GROUP” by OrthoMCL due to the best matching proteins not having a group in the database. We found 15,668 common orthologs between the two dataset, while 9,591 and 12,366 orthologous groups were unique to the NN and NO datasets, respectively (these accounted for 38.0% and 44.1% of the respective datasets) (**Figure 3E** and **Supplementary Table 3**).

### More Long Noncoding Transcript Isoforms Are Detected in the Normalized Library

It is suggested from analysis by TransDecoder above that there were more transcripts that did not exhibited an ORF in the NO dataset (16% sequences) compared to the NN dataset (7% sequences). We asked whether the normalization recovered more long noncoding (lnc) transcripts since the lnc transcripts are generally shorter and expressed at a lower level than that of coding transcripts (Wang et al., 2015; Golicz et al., 2018). We first extracted the transcripts that did not exhibit any ORF (min 100 aa) and calculated the coding potential using CPC ver0.9-r2 against the UniRef90 protein database. Those transcripts with a CPC score <0 were classified as lnc transcripts and compared between the two datasets. The results showed that 932 sequences (N50: 1,137 nt) and 4,416 sequences, (N50: 928 nt) were predicted as “non-coding” from the extracted sets of NN and NO datasets, respectively. About 79.9% (3,527 sequences) of the total predicted lnc transcripts in the NO dataset belonged to the unique fraction of the NO dataset **(** **Figure 3F**), which was generally expressed at a lower level than that from the NN dataset (see the next section for transcript expression analysis). Of 932 lnc transcripts from the NN dataset, 692 sequences (74.2%) were found in the unique fraction of the NN dataset. The result implies that the normalization recovered more number of lnc transcripts that were expressed at a lower level compared to coding genes. Some of NN lnc transcripts could be those longer transcripts that were removed by the normalization process or those were expressed a relatively higher level, while those that were detected only in the NO dataset could be those were lowly represented and not sequenced in the NN dataset.

### Short-Read Mapping Using Read Data From Different Tissue Types Reveals a Fraction of Lowly Expressed Transcripts in the Normalized Library

**Figure 4:**
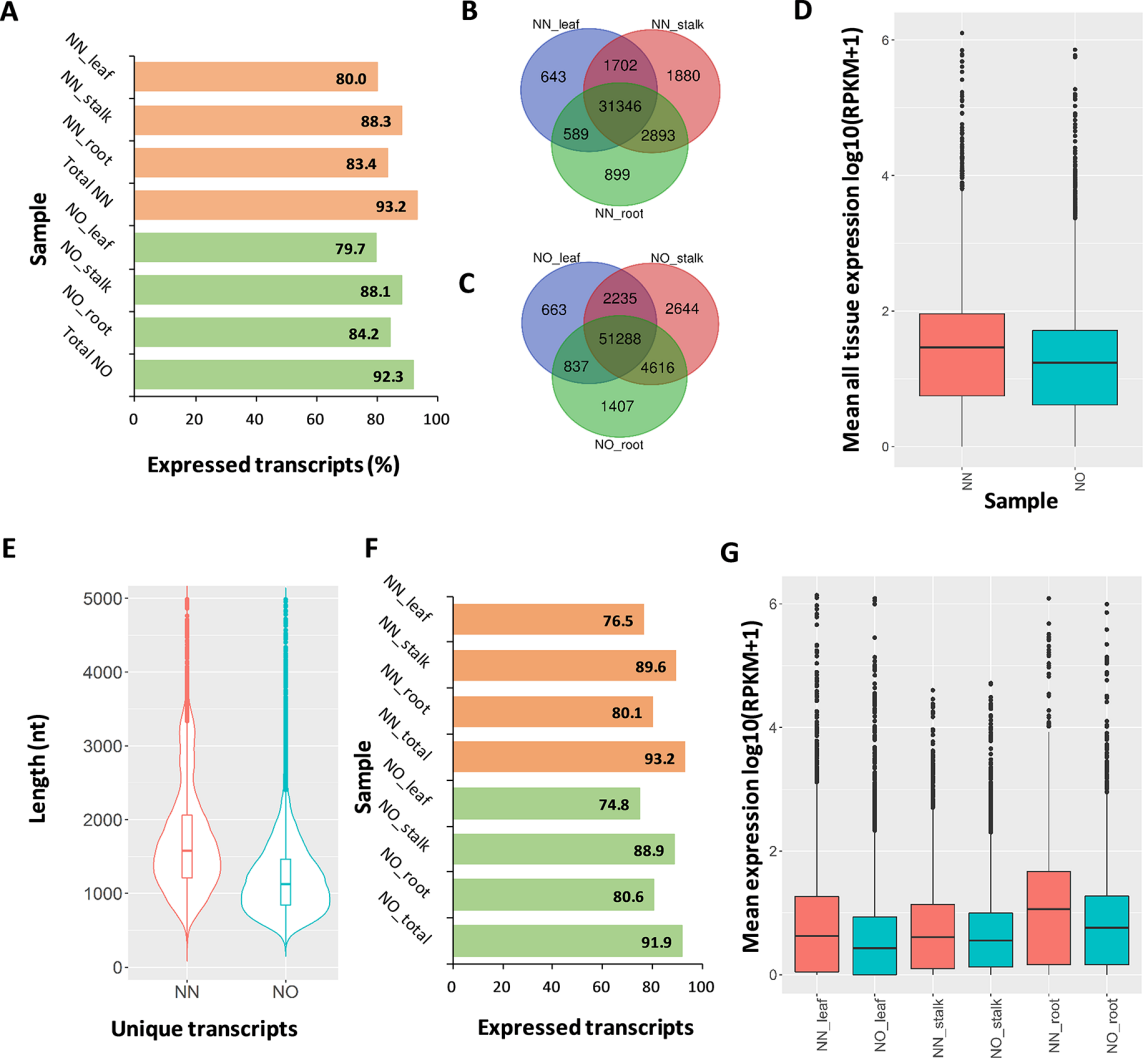
Expression analysis. **(A)** Percentage of expressed transcripts from the NN and NO datasets detected in three different tissues: leaf, stalk, and root. **(B)** Comparison of expressed transcripts in each tissues expressed in the NN dataset. **(C)** Comparison of expressed transcripts in each tissues expressed in the NO dataset. **(D)** Mean expression level across all three tissues of the NN and NO datasets. The expression level (RPKM) was log10 transformed for visualization purpose. **(E)** Length distribution of the two fractions of unique transcripts in the NN and NO datasets. **(F)** Percentage of expressed unique transcripts from the NN and NO datasets detected in three different tissues: leaf, stalk and root. **(G)** Comparison of expression level between unique transcripts from the NN and NO datasets, across three tissues: leaf, stalk and root. The expression level (RPKM) was log10 transformed for visualization purpose. NN, sugarcane non-normalized PacBio Iso-Seq isoforms; NO, sugarcane normalized PacBio Iso-Seq isoforms.

We validated the expression of the transcript isoforms of the two datasets by mapping RNA-Seq data representing leaf, root tissues (Mason et al., unpublished), and stalk tissue (Hoang et al., 2017b) against the two transcript datasets (**Supplementary Table 4**). The analysis revealed that 93.2% of NN transcripts and 92.3% of NO transcripts were supported by the short-read data (RPKM >0 in either tissues) (**Figure 4A**). Using the RNA-Seq data from leaf, stalk, and root tissues, we found that more transcript isoforms were validated using the stalk tissues, compared to that from root and leaf tissues (**Figures 4B, C**). The majority of validated transcripts were found to be expressed in all three tissue types, while there were more unique transcripts expressed in the stalk and root tissues, in both datasets. Since the PacBio data were generated from a pooled RNA sample of 22 genotypes, using a larger RNA-Seq data set from different varieties would be likely to validate more transcript isoforms. When the mean expression level of each transcript across three tissues was compared, generally, it was revealed that the expression level in NN dataset was higher than that of the NO data set (**Figure 4D**).

The aim of transcript normalization prior to sequencing was to reduce the abundance of the very highly expressed transcripts, so that the lowly expressed transcripts could be represented and sequenced, and thereby, to increase the representation of the transcriptome. A higher representation of the transcriptome was shown by the higher total transcript isoform number, higher BUSCO completeness, and greater number of gene families and ortholog counts for the NO dataset, compared to the NN dataset, in the previous sections. We tested the hypothesis of whether the NO dataset recovered more lowly expressed transcripts by mapping the RNA-Seq read data from three tissue types (leaf, stalk, and root) against the two unique fractions of NN and NO datasets identified in **Figure 2B** and used the mean expression value for comparison. The total unique transcripts was 22,566 (N50: 1,864 nt) and 36,074 (N50: 1,324 nt) for the NN and NO datasets, respectively (**Figure 4E**). The expression level of each transcripts across three tissue types was analyzed. Overall, about 77–90% of the total 22,566 unique transcripts from the NN dataset were found to be expressed, while ∼75–89% of the total 36,074 transcripts from the NO dataset were expressed (**Figure 4F**). The expression of the unique transcripts from the NO dataset was generally lower than that of the unique transcripts from the NN dataset, for all three different tissues tested (**Figure 4G**). Furthermore, the expression difference between the two unique transcript fractions could be observed more clearly in the leaf and root tissues, compared to that in stalk tissues. Collectively, the results suggest that normalization led to more lowly expressed transcripts being sequenced, and there was likely to be a proportion of lowly expressed transcripts that were not detected using the RNA-Seq data, which could require a larger read data to be validated.

### Each Library Recovers a Unique Fraction of Functional Transcripts

The transcripts in the two datasets represented only polyadenylated (polyA) RNAs in the sugarcane transcriptome, since oligo(dT) was used in our RNA extraction protocol. PolyA transcripts could be protein-coding (mRNAs) or noncoding transcripts (Liu et al., 2015). We have predicted earlier that a higher transcript percentage in the NN dataset exhibited ORFs (min 100 aa) potentially encodes proteins than that in the NO dataset (93% vs. 84%), and only 2.2% (932 transcripts) in the NN and 6.4% (4,416 transcripts) in the NO dataset were long noncoding. The un-categorized transcripts could include those that encode small peptides (min <100 aa), regulatory transcripts, and un-annotated transcripts (Chew et al., 2013; Jabnoune et al., 2013; Ruiz-Orera et al., 2014).

**Figure 5:**
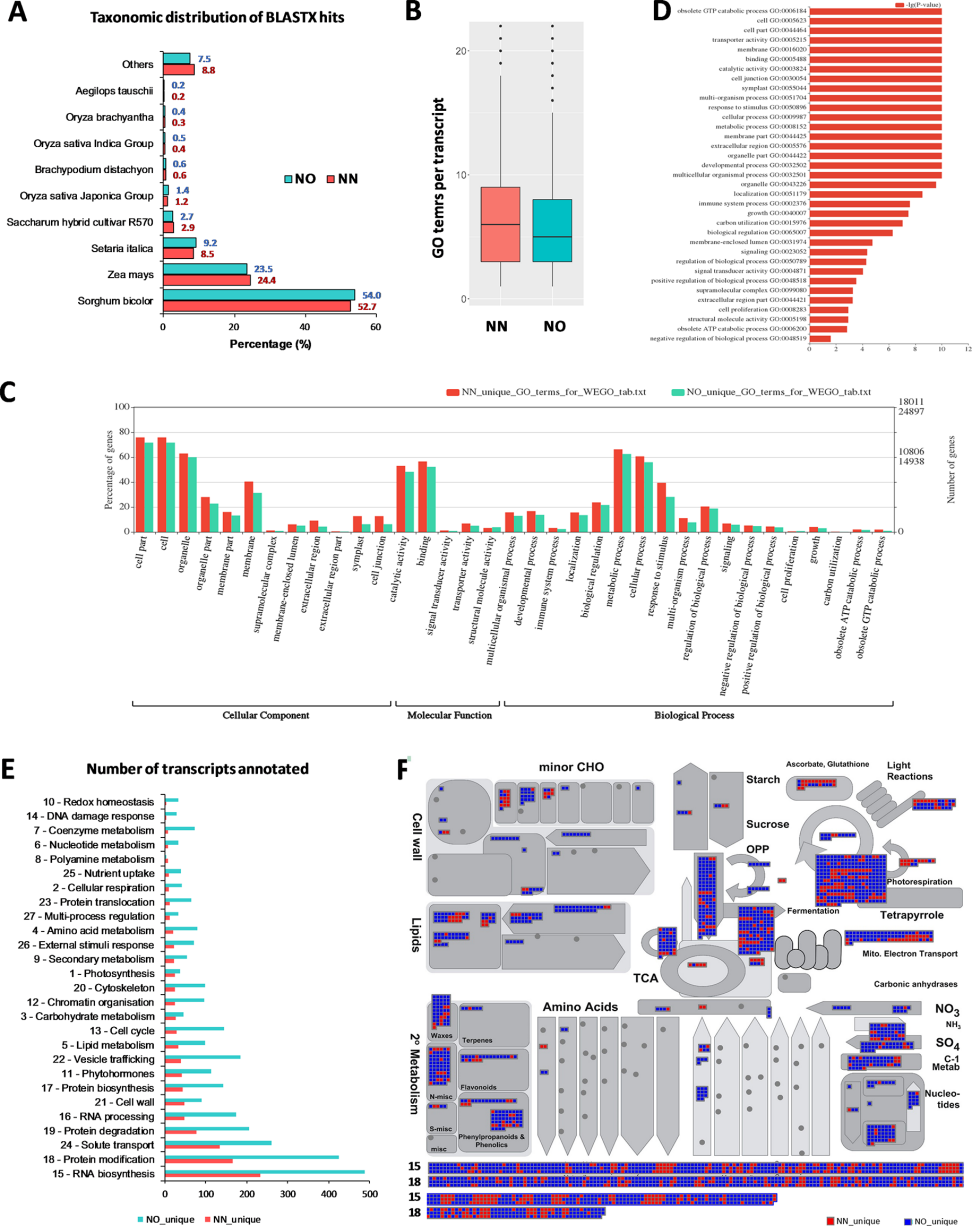
Functional annotation of transcript isoforms from the two datasets. **(A)** Taxonomic distribution of BLATX hits of the two datasets. **(B)** GO terms per transcripts. **(C)** Significantly different GO terms between the two datasets. **(D)** Significantly different GO terms with highest log_10_(p value) identified from the two datasets. **(E)** Unique bins annotated using *Arabidopsis* genes that matched the unique fractions of the NN and NO datasets. **(F)** MapMan functional bins identified from the two datasets, red heatmap represents transcripts from the NN dataset, while blue represents those from the NO dataset. Two bins (15 and 18) were added to the bottom of the panel listing all matched genes from two datasets. Each heatmap represents one annotated transcript. NN, sugarcane non-normalized PacBio Iso-Seq isoforms; NO, sugarcane normalized PacBio Iso-Seq isoforms.

To gain further insights into the difference between the transcripts captured in the two libraries, we annotated and compared transcripts using KOBAS ver3.0 and FunctionAnnotator. We were able to annotate 38,232 out of 42,845 transcripts (∼89% of the NN dataset), and 57,699 out of 69,022 transcripts (∼84% of the NO dataset) using KOBAS against the complete genome of *Sorghum bicolor* with an e-value of 1e-5. The number of unique sorghum genes identified were 10,544 and 14,069 genes, respectively, for the NN and NO datasets, totaling 15,749 unique sorghum genes identified (**Supplementary Table 5**). Using the NCBI NR protein database through FunctionAnnotator, 95% (40,631 sequences) and 90.5% (62,443 sequences) of the NN and NO datasets were found as hits, while ∼84% NN dataset (36,103 sequences) and 80% NO dataset (53,820 sequences) were found mapped to 90,499 and 130,906 GO terms, respectively (**Table 2**). In relation to the taxonomic distribution of BLASTX result hits, *S. bicolor*, *Zea mays*, and *Setaria italica* were found to be the top three species (**Figure 5A**). In all cases, there were more genes identified, yet a higher proportion of unique transcripts left un-annotated in the NO dataset, which could be attributed to the higher proportion of non-coding transcripts or transcripts encoding unknown proteins in the NO dataset. The results are consistent with the higher percentage of coding transcripts (ORF of minimum 100 aa) predicted in the NN dataset by TransDecoder, and more lnc transcripts identified in the NO dataset by CPC. It is important to note here that, even though the NO dataset mapped to more GO terms, the number of GO terms assigned per transcript was lower than that for the NN dataset (mean 6.1 *vs*. 7.1, respectively) (**Figure 5B**), suggesting that there were more transcripts belonging to several GO groups in the NN dataset. This is likely to be due to the longer length of transcripts in the NN dataset compared to that in the NO dataset (as shown earlier in **Figure 2A**). However, when extracted GO terms were compared by the Pearson chi-square test (proportion-based) with cutoff p-value <0.05 through WEGO 2.0, there were no significant GO term enrichment between the two total datasets.

**Table 2:** Functional annotation summary of the two datasets used in this study.

	NN dataset	NO dataset	NN_unique	NO_unique
**Total sequences**	42,845	69,022	22,566	36,074
**NCBI_NR protein hits**	40,631	62,443	20,742	30,391
**NR percentage (%)**	**94.8 **	**90.5 **	**91.9 **	**84.2 **
**GO annotated**	36,103	53,820	18,011	24,897
**GO percentage (%)**	**84.3 **	**78.0 **	**79.8 **	**69.0 **
**Biological process terms**	30,579	43,786	15,356	19,828
**Cellular component terms**	31,159	45,744	15,255	20,270
**Molecular function terms**	28,761	41,376	14,741	19,038
**Total GO terms**	90,499	130,906	45,352	59,136

Next, we looked into the two unique fractions from the NN and NO datasets (22,566 and 36,074 transcripts, respectively) identified by CD-HIT-EST-2D by comparing their functional enrichment. Similar to the total sets, there was a higher proportion of un-annotated transcripts in the unique NO compared to that in the NN dataset. About 92% (20,742 sequences) and ∼84% (30,391 sequences) for the two unique fractions from NN and NO datasets were mapped against the NCBI NR protein database, of which 18,011 sequences and 24,897 sequences were assigned to 45,352 and 59,136 GO terms, respectively (**Table 2**). Using the extracted GO terms, we compared the two datasets by the Pearson chi-square test with a cutoff p-value <0.05 in WEGO. **Figure 5C** presents only the GO terms belonging to the three categories, cellular component, molecular function, and biological process that were significantly different between the two unique fractions at a p-value <0.05, and **Figure 5D** shows the most significant GO terms based on their -log_10_ (p-value). Most of the significantly different GO terms showed a higher percentage in the NN unique dataset, compared to that of the NO unique faction. Since WEGO compared GO distribution using percentage of transcripts within the total annotated transcripts, the difference could be attributed to the lower GO terms per transcript in the NO dataset compared to that in the NN dataset (5.6 vs 7.1 GO terms per transcripts, respectively), and probably the distinct functional roles of transcripts from each unique fractions.

Further functional differences were analyzed by Mercator 4 using *Arabidopsis* gene IDs obtained in transcript annotation using KOBAS. About 83.2% (35,655 sequences mapped to 9,253 *Arabidopsis* genes) of the NN dataset and 75.1% (51,820 sequences mapped to 11,679 *Arabidopsis* genes) of the NO dataset were annotated. Using the *Arabidopsis* gene IDs, we identified 1,499 and 3,925 *Arabidopsis* genes that were only found of the NN and NO datasets, respectively. We parsed these unique gene sets using the Mercator pipeline and compared their functional annotation. **Figure 5E** presents the total number of transcripts mapped to the MapMan bins from the NN and NO datasets, with more transcripts found in the NO dataset. Amongst the bins, RNA biosynthesis (bin 15) and protein modification (bin 18) included most transcripts in both dataset. The overview of annotation and MapMan functional bins are shown in **Figure 5F**, with clear differences in the number of transcripts annotated for each of the datasets, and many sub-bins where only transcripts from the NO were found (blue heatmaps). Within bin 15 (RNA biosynsthesis, lower panel **Figure 5F**), many transcription regulators were found unique to each dataset (**Supplementary Table 6**). These transcription regulators belonged to several transciption factor (TF) families including TFIId basal transcription regulation complex, TATA box-binding protein (TBP) regulation, SAGA transcription co-activator complex, MEDIATOR transcription co-activator complex, DCL1-HYL1 miRNA biogenesis complex, TFIIIc transcription factor complex, C2C2 superfamily, E2F/DP transcription factor, EIL (EIN3-like) transcription factor, GRAS transcription factor, HSF (heat shock) transcription factor, MADS box transcription factor, C2H2 zinc finger transcription factor, C3H zinc finger transcription factor, NAC transcription factor, SBP transcription factor, MYB superfamily, WRKY transcription factor, HB (homeobox) superfamily, PHD finger transcription factor, bHLH transcription factor, bZIP superfamily, B3 superfamily, AP2/ERF superfamily, and mTERF transcription factor. Similarly, in the functional bin 18 (protein modification, lower panel 4F), there were several genes only found in one of the datasets (**Supplementary Table 7**). These included transcripts encoding DPMS dolichol-phosphate-mannose synthase complex, dolichol-phosphate-linked oligosaccharide precursor assembly, oligosaccharyl transferase (OST) complex, complex N-glycan maturation, and GPI pre-assembly. GPI N-acetylglucosamine transferase complex, GPI pre-assembly, TKL kinase superfamily, STE kinase superfamily, CMGC kinase superfamily, CK kinase superfamily, CAMK kinase superfamily, AGC kinase superfamily, atypical kinase families, phosphorylation, dephosphorylation, S-glutathionylation and deglutathionylation, protein folding and quality control, and peptide maturation.

Taken together, the results suggest that the two datasets included different functional transcripts of the studied sugarcane transcriptome, in addition to the common transcripts presented in both libraries. The NN captured a wider length range of transcripts and had a lower number of transcripts resulting from deeper sequencing of highly represented sequences and a higher percentage of sequences that were annotated using the known protein database and GO database. This datas*et al*so possessed more transcript isoforms per gene family and GO terms per transcripts. The NO dataset, on the other hand, included more transcript isoforms, of a shorter length, representing a higher transcriptome completeness and more gene families, and had generally lower expression levels and more un-annotated transcripts using the known protein databases. Many of the NO transcripts were found to be long noncoding transcripts, mostly derived from the unique NO fraction, and were expressed at a lower level than protein-coding transcripts.

### Distribution of the Transcript Isoforms on the Reference Genomes

**Figure 6:**
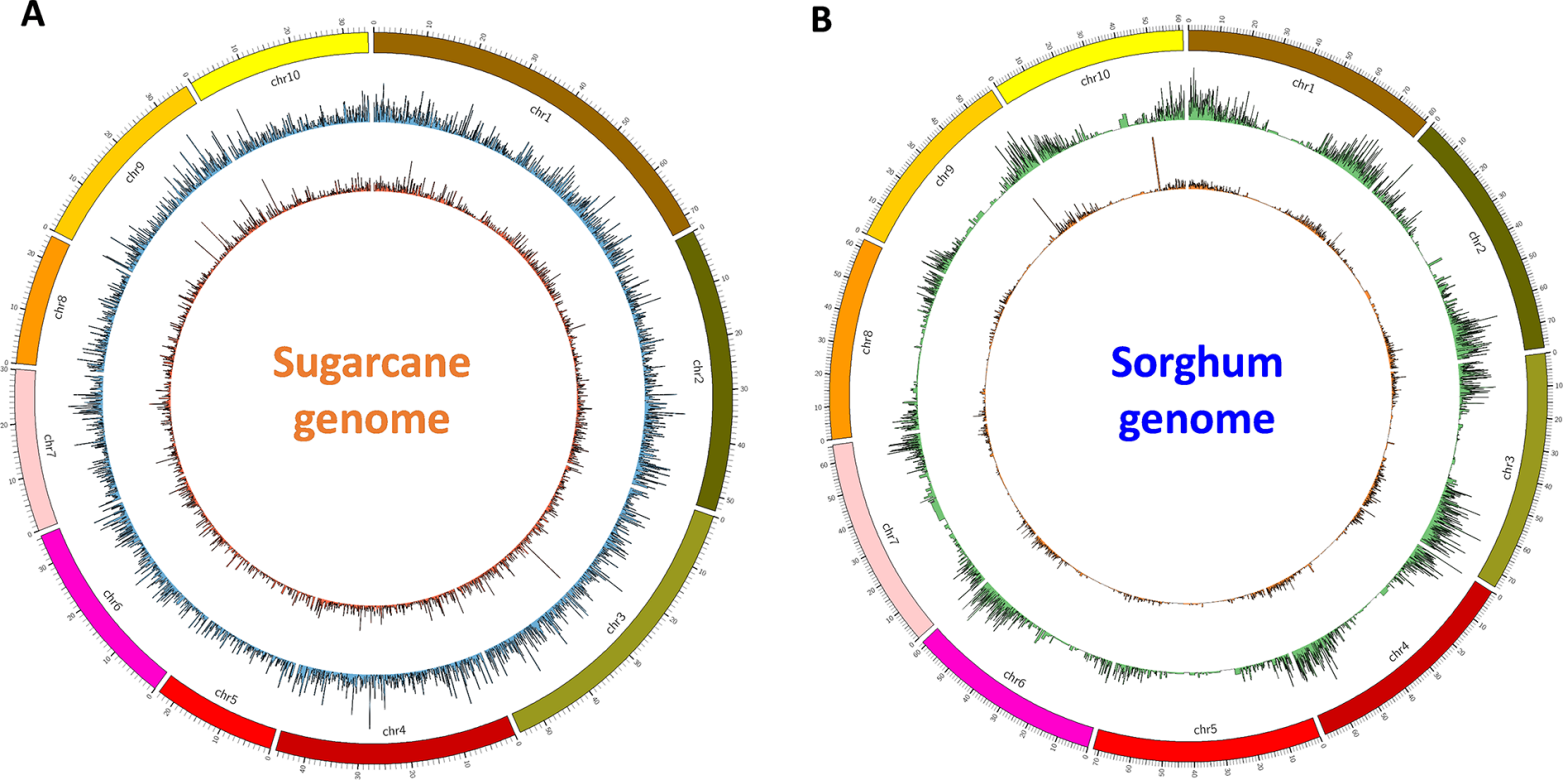
Transcript isoform distribution on the sugarcane genome **(A)** and the sorghum genome **(B)** using Circos. Outer graphs present mappable transcripts from the NO dataset, while the inner graphs present mappable transcripts from the NN dataset on the genomes. NN, sugarcane non-normalized PacBio Iso-Seq isoforms; NO, sugarcane normalized PacBio Iso-Seq isoforms.

Finally, for validation and visualizing of transcript distribution along the reference genome, we mapped all transcripts from NN and NO datasets against the newly released sugarcane monoploid genome sequences (Garsmeur et al., 2018) using the splice-aware mapper GMAP program at 80% coverage and 80% identity threshold. It was found that the NN dataset had a slightly higher percentage of reads mapped to the sugarcane monoploid genome compared to the NO dataset, being 61.5% and 59.3%, respectively **(** **Figure 6A**). Using the same settings for read mapping against the sorghum genome sequences ver3, it was 75.5% and 70.5% mapped transcripts from NN and NO, respectively **(** **Figure 6B**). Most of transcripts were distributed more evenly across the monoploid sugarcane genome, while in the case of sorghum genome, the transcript distribution was biased toward the chromosome arms and less in the centromeres.

## Discussion

Normalization of cDNA could potentially result in a reduction of sequencing time, computational effort, and cost in obtaining a high-quality and complete transcriptome using the currently available sequencing platforms. It is known that, within the mRNA population, about 20% of the total transcript copies are attributed to a few genes (5–10 genes); 40–60% are from 500–2,000 genes; while the rest 20–40% are originally from low/rare transcripts (Alberts et al., 1994). This vast difference in transcript expression level normally leads to the highly expressed transcripts being recurrently sequenced multiple times, while those rare transcripts might be excluded depending on the sequencing depth. This might result in a better quality for those highly abundant transcripts by facilitating more efficient error correction using their high sequencing coverage; it might also result in a low number of genes being detected. To obtain a high-quality and more representative transcriptome, a high coverage of sequencing is required for a non-normalized cDNA library to ensure that more medium and lowly expressed transcripts are captured (Abdel-Ghany et al., 2016; Wang et al., 2016). To avoid unnecessary sequencing of the redundant sequences and increase the gene discovery rate by equalizing the transcript abundance, a cDNA normalization method employing DSN enzyme isolated from Red King (Kamchatka) crab was developed (Zhulidov et al., 2004; Zhulidov et al., 2005; Bogdanov et al., 2010; Bogdanova et al., 2011). The method has been applied in enrichment and construction of normalized RNA-Seq libraries for second-generation sequencing, including 454 and Illumina (Fox et al., 2009; Lai et al., 2012). The reported reduction of transcript abundance could be up to 1,000-fold (Anisimova et al., 2006).

In recent years, the third-generation of sequencing technologies has been emerging as a powerful tool to recover the complexity of transcriptomes without the need of assembly and provide more accurate evidence of transcript length, alternative splicing events, and polyadenylation sites. There are a growing number of plant transcriptomes that have been sequenced by long-read technologies, which, in turn, facilitated the reference genome annotation and isoform-level transcript profiling (Dong et al., 2015; Zhichao et al., 2015; Abdel-Ghany et al., 2016; Wang et al., 2016; Cheng et al., 2017; Clavijo et al., 2017; Hoang et al., 2017a; Li et al., 2017b; Mascher et al., 2017; Wang et al., 2017a; Wang et al., 2017b; Wang et al., 2018; Workman et al., 2018). cDNA normalization was employed for many of these studies with the purpose of detecting more genes and rare transcripts, while deep sequencing of a non-normalized cDNA library was also used in many others, aiming to render normalization largely unnecessary. This study set out to evaluate the impact of cDNA normalization on long-read sequencing using the complex transcriptome from the highly polyploid sugarcane genome as a test case. We exploited a full-length transcriptome data set recently generated by PacBio Iso-Seq technology derived from two cDNA libraries of 22 sugarcane cultivars, with and without cDNA normalization (Hoang et al., 2017a). The results highlighted the quality-related differences between the two libraries and pinpointed the pros and cons of normalization in the context of long-read sequencing of transcriptomes.

By using the highly polyploid sugarcane transcriptome to test the impact of the normalization on long-read sequencing by PacBio Iso-Seq, we showed that, besides many of the same transcripts being captured, normalization removed many longer transcripts, while include many new generally shorter transcripts. The normalized library recovered more transcript isoforms (69,022 transcripts) in total compared to the non-normalized library (42,845 transcript sequences). Of the total transcripts, 93.2% of the non-normalized transcripts and 92.3% of the normalized transcripts were supported by the Illumina short-read data of different tissues including leaf, stalk, and root tissues. Although medium-sized Illumina RNA-Seq data (maximum sample reads were ∼66 million) was used to confirm the expression of transcript, the use of deeper coverage data is likely to confirm more transcripts, especially those lowly expressed in the normalized dataset. Moreover, 75.5% and 70.5% of the transcripts from respective non-normalized and normalized datasets were aligned against the sorghum genome version 3, while 61.5% and 59.3% aligned to the new monoploid sugarcane genome. Transcripts aligned evenly across the sugarcane chromosomes, but mostly on the two arms toward the telomeres on the sorghum chromosomes. The lower percentage of transcripts mapping against the sugarcane monoploid genome could be due to the genome sequences being derived from a French cultivar R570 representing only the sugarcane gene space that covered the sorghum genome (Garsmeur et al., 2018). It could also be that our transcriptome data were generated from Australian cultivars which could have distinct genetic architectures. The genetic composition and chromosome number of sugarcane hybrids are known to be unique to each cross, due to the random chromosome sorting and recombination of the two parental lines used for hybridization (Grivet and Arruda, 2002). A lower percentage of the transcripts from the normalized library mapped against the reference genome compared to that of the non-normalized library, possibly because this library had more transcripts that come from the genome regions that are not represented by the reference genome sequences of sugarcane or sorghum.

When compared, the two libraries had about 47.3% and 47.7 of sequences that were common (at 80% identity), while the rest (equivalent to 22,566 non-normalized transcripts and 36,074 normalized transcripts) were unique to one of the libraries. Similar to the results for the total sets, the unique normalized transcripts exhibited shorter length compared to the unique non-normalized transcripts. Analysis revealed that the unique non-normalized transcripts were expressed at a higher level than the unique normalized transcripts. This suggested that the normalization recovered a proportion of lowly expressed transcripts that were likely to be absent in the non-normalized library. The unique non-normalized transcripts could be those long transcripts that were removed by the normalization due to its bias toward shorter transcripts. Among the unique normalized transcripts, we identified 3,527 long-noncoding transcripts, accounted for ∼9.8% of the unique sequences and ∼79.9% of the total 4,416 long non-coding transcripts found in the normalized library. Only 932 long non-coding transcripts were found in the non-normalized library, of which, 692 sequences (∼3% total unique transcripts and ∼74.2% total long-noncoding transcripts in the non-normalized dataset) were in the unique fraction. Therefore, the total number of long non-coding transcripts identified in this study were 5,348, more than the total of ∼2,400 sequences reported previously in (Hoang et al., 2017a) using homology searching and coding prediction. Here, a more systematic approach using the CPC package against the UniRef90 protein database for long noncoding transcript identification was conducted. The lower expression of normalized long non-coding transcripts compared to protein-coding transcripts is in agreement with several reports (Wang et al., 2015; Golicz et al., 2018). Further functional annotation against know protein databases indicated that the non-normalized dataset had a higher percentage of annotated transcripts compared to the normalized dataset. This could be that, besides more long noncoding transcripts, the normalized library is likely to include more transcripts that were not detected in previous experiments due to their low expression level (that were normally excluded in the library preparation).

In terms of representing the sugarcane transcriptome, the normalized library was found to have a better transcriptome completeness, by including 86.2% of the eukaryotic BUSCOs, compared to 75.3% found in the non-normalized library. This result is consistent with the number of gene families and orthologous groups predicted, in which the normalized library had 9,783 putative gene families; 28,034 orthologous groups compared to 5,818 gene families; and 25,259 orthologous groups in the non-normalized library. Using the combined data from non-normalized and normalized libraries led to an improved performance, in which 92.7% BUSCO completeness; 13,276 gene families; and 37,625 orthologous groups were detected. This suggests that sequencing of the two libraries could result in more gene families being detected, including the rare transcripts. This probably could be achieved by sequencing at a sufficiently deep coverage of a non-normalized library; however, this would result in the highly abundant transcripts being overrepresented in the pre-processed data. In general, our results are in line with several reports based on earlier short-read sequencing platforms (454 and Illumina), in which a higher rate of gene discovery was found in the normalized library (Ewen-Campen et al., 2011; Ekblom et al., 2012; Mroz et al., 2018). It is noteworthy that the sequencing of a merged library of non-normalized and normalized cDNA was employed in many studies to include more genes (Karako-Lampert et al., 2014; Meireles et al., 2018). It is also important to note that even though normalization of cDNA using DSN reduces the time of sequencing of the same transcripts, and thereby reduces the cost and computational effort, it could suffer from the length bias toward removing longer transcripts and bias toward AT-rich transcripts, and hence against the transcripts with high GC content (Matvienko et al., 2013; Boone et al., 2018). It is probably ideal to sequence a non-biased normalized library in combination with size fractionation to include expected length range and transcripts of different expression level. Optimizing the normalization protocol would be the best strategy for sequencing of a transcriptome where we expect transcripts are expressed at hugely variable levels. Specific bioinformatics tools including TAPIS pipeline (Abdel-Ghany et al., 2016), *de novo*AS (Liu et al., 2017), IsoCon (Sahlin et al., 2018), and isONclust (Sahlin and Medvedev, 2019) could be used to process and cluster read data prior further functional annotation at transcript isoform level.

Finally, sugarcane is an important industrial crop with a great potential for biofuel and biomaterial production to reduce our dependence on fossil fuel energy. Modification of sugarcane biomass can be tailored by genetic approaches for a better composition for converting to biofuels and other high value molecules (Waclawovsky et al., 2010; de Souza et al., 2014; Furtado et al., 2014; Hoang et al., 2015; Kandel et al., 2018). The sugarcane genome is complex, and this hinders the sequencing progress and understanding of the genome structure and functions, compared to progress made for other grass species including sorghum and maize in recent years (Souza et al., 2011; Thirugnanasambandam et al., 2018). The newly constructed transcriptome (SUGIT, which was based on the data used in this study) has been shown to be useful in assessing short-read transcriptome assemblies (Hoang et al., 2018), transcript profiling to identify differentially expressed transcripts (Hoang et al., 2017b; Marquardt et al., 2018; Thirugnanasambandam et al., 2017), reconstruction of highly synthetic genes between sugarcane and related species (Mancini et al., 2018; Thirugnanasambandam et al., 2019), and annotation of the sugarcane progenitor genome (Zhang et al., 2018). More recently, the genome sequences for sugarcane species have been released for different cultivars including the Brazilian cultivar SP32-8032 (Riaño-Pachón and Mattiello, 2017), the French cultivar R570 (Garsmeur et al., 2018), as well as, for the autopolyploid parental species *Saccharum spontaneum* (Zhang et al., 2018). As part of this study, we have further processed the PacBio transcriptome data by removing any contamination sequences from adapters or primers to make it more usable. This clean full-length transcriptome data would aid in improving annotation of functionally expressed genes in the genome by providing direct transcript evidence.

To conclude, our work represents a comprehensive analysis of the impact of cDNA normalization on the long-read sequencing using the PacBio Iso-Seq read data. The analysis suggested that while the two libraries included many of the same transcripts, many longer transcripts were removed by normalization, and many new generally shorter transcripts were detected. The non-normalized library included less transcript isoforms of those highly represented in the sugarcane transcriptome and resulted in a higher percentage of annotated transcripts against known protein databases. The normalized library recovered more total transcript isoforms, representing more predicted gene families/orthologous groups and a higher completeness of the sugarcane transcriptome. There was a higher percentage of un-annotated transcripts in the normalized library, suggesting that this library potentially includes more novel transcripts which were not in current transcript databases. This un-annotated fraction could be long non-coding transcripts, regulatory transcripts, and transcripts that encode small peptides. A total of 5,348 long noncoding transcripts were predicted in this study, of which, ∼83% was derived from the normalized library. A large proportion of the unique transcripts accounted for more than half of the normalized library including ∼80% of its predicted long noncoding transcripts were expressed at a lower level than that of the unique transcripts from the non-normalized library, across three different tissue types tested including leaf, stalk, and root. Functional annotation of the two unique transcript fractions suggested that many of the important transcripts were detected in both datasets. The results demonstrated the complementation of the two approaches in obtaining a complete transcriptome of a complex genome, at the given sequencing depth.

## Author Contributions

RH and AF conceptualized the study. RH, AF, and NH designed the experiments. NH, AF, and VP conducted the analysis. RH, FB, AF, VP, and NH discussed the results. NH prepared the first draft. RH, FB, AF, VP, and NH critically revised the manuscript. All authors discussed the results and read and approved the final manuscript.

## Funding

This work was funded by the Queensland Government and Sugar Research Australia (SRA). "SRA" had no role in study design, data collection and analysis, decision to publish, or preparation of the manuscript. We are grateful to the Australian Agency for International Development (AusAID) for financial support through an Australian Awards Scholarship to NH.

## Conflict of Interest Statement

Author FB was employed by the company, Sugar Research Australia. All authors declare that the research was conducted in the absence of any commercial or financial relationships that could be construed as a potential conflict of interest.
